# Long Segment Urethral Reconstruction Using a Prefabricated Hairless Groin Skin Flap Based on the Descending Branch of Lateral Circumflex Femoral Artery

**DOI:** 10.1055/s-0046-1817013

**Published:** 2026-04-15

**Authors:** Mohit Sharma, Shikha Gupta, Vasundhra Jain, Srilekha Reddy G., Devajyoti Guin, Anil Murarka

**Affiliations:** 1 Department of Plastic and Reconstructive Surgery, Amrita Hospital, Faridabad, Haryana, India

**Keywords:** urethral reconstruction, prefabrication, hypospadias

## Abstract

Congenital or acquired anomalies of the penis can lead to significant physical and psychological problems in a patient. Goals of urethral reconstruction is to create a functional meatus with a patent tube for micturition and ejaculation. In a patient with penoscrotal hypospadias we reconstructed 13 cm length of the urethra using a two-stage procedure. In the first stage, we prefabricated a flap using the hairless groin skin with the descending branch of the lateral circumflex femoral artery. In the second stage, the flap was transposed and tubed to create the neourethra. Long segment urethral reconstruction using prefabricated hairless groin skin flap based on the descending branch of lateral circumflex femoral artery is a new technique of near-total urethral reconstruction to the best of our knowledge and can be used to treat difficult cases of urethral reconstruction for varied indications, giving us a simple and innovative technique in reconstructive armamentarium.

## Introduction

Congenital or acquired anomalies of the penis can lead to significant physical and psychological problems in a patient. 1 Goals of hypospadias correction is to create an aesthetic phallus with a functional meatus with a patent tube for micturition and ejaculation. Urethral reconstruction in failed hypospadias repair is difficult due to the paucity of the available tissue. In these cases, the ideal option for reconstruction is a vascularized flap. 2 Various methods for long urethral reconstruction have been described using buccal mucosa, colonic mucosa, jejunal flap, fasciocutaneous ALT flap, and radial artery forearm flap; out of these, the radial artery forearm flap is most commonly performed. We have used a prefabricated flap of hairless groin skin based on the descending branch of lateral circumflex femoral artery (LCFA) for reconstructing the urethra in a staged procedure.

## Case Report

**Fig. 1:**
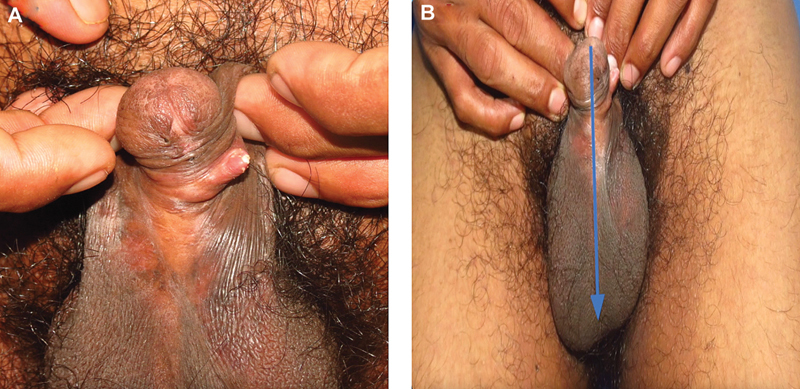
( **A** ) Preoperative image. ( **B** ) Preoperative image demonstrating healthy urethra at penoscrotal junction (blue arrow).

Our patient was a hypospadias cripple with many fistulas operated multiple times. On dissection the healthy urethra was available only at the penoscrotal junction. The length of urethral reconstruction required to bring the urethral meatus till glans tip was 13 cm. This was determined by creating an artificial erection during the procedure and the length measured from the penile tip to the urethral opening was found to be 13 cm ( Fig. 1 ).

We decided to prefabricate a neourethra from the hairless groin skin by utilizing the descending branch of LCFA with its vena commitantes.

### Surgical Technique

**Video 1** ( **A, B** ) Demonstration of the surgical technique and the postoperative outcome.


**Fig. 2:**
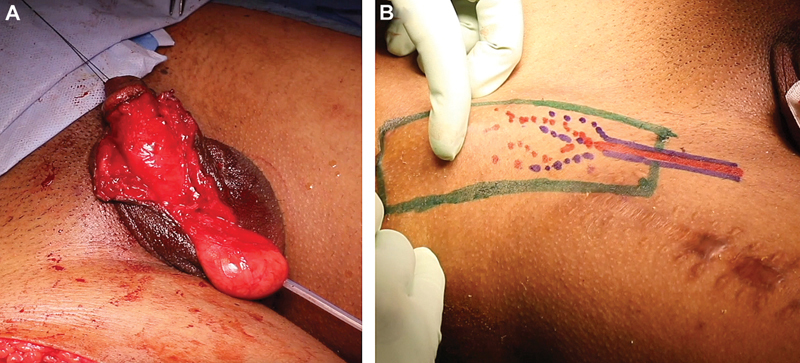
( **A** ) Recipient dissection to expose the penile shaft and to assess the site of healthy native urethra. Length of urethra required was 13 cm. ( **B** ) Flap marking for stage one—prefabrication of the flap. Flap measuring 13 × 3 cm was planned with descending branch of lateral circumflex femoral artery (LCFA) pedicle sutured under the flap covered with silicone sheet.

Stage I: A thin groin skin pocket was created in the subcutaneous plane, and the dissected vascular pedicle (descending branch of LCFA) was sutured under the skin with 7–0 prolene stitches. A silicone sheet was placed underneath the skin and the vascular pedicle. Another silicone sheet was used to isolate the pedicle itself. The groin skin where the prospective urethra would be planned was marked at its four corners with 5–0 prolene stitches for future identification of the site. This marked the completion of first stage of operation. Penoscrotal level urethral opening was kept catheterized ( Figs. 2 3 4 ).

**Fig. 3:**
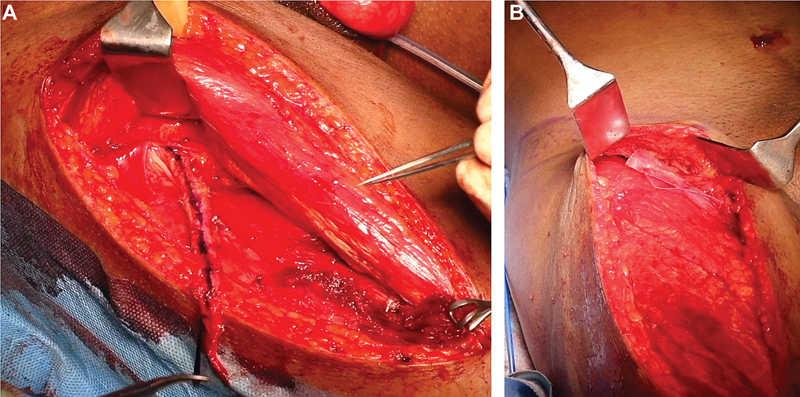
( **A** ) Descending branch of lateral circumflex femoral artery (LCFA) pedicle harvested from the right thigh. ( **B** ) Groin skin pocket creation done. LCFA pedicle transferred under the skin and sutured with 7–0 prolene and covered with silicone sheet.

**Fig. 4:**
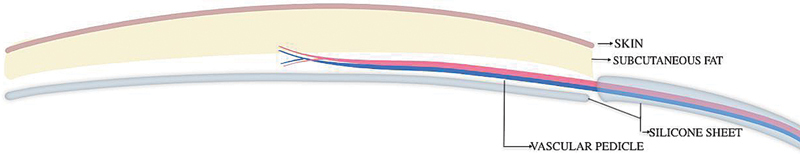
Diagrammatic representation of the prefabricated pedicle and the placement of the silicone sheets.

The second stage was performed after 4 months.

**Fig. 5:**
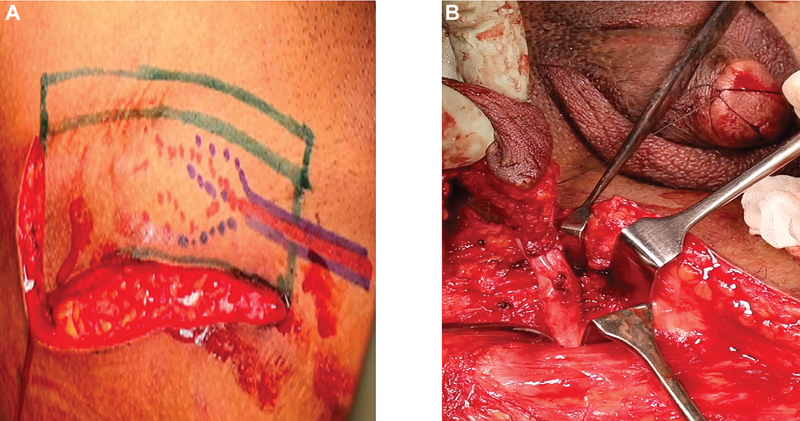
( **A** ) Flap marking in stage II with the position of pedicle as per the Doppler. ( **B** ) Pedicle dissection after flap elevation.

Stage II: A glans stay stitch was placed and the vessels and prospective urethral skin was marked based on the previous suture markings. The newly vascularized prefabricated flap was raised, and underlying silicone sheet was removed. The vascular pedicle was carefully dissected. The flap and the pedicle were completely separated from the overlying skin, and the pedicle was marked and the flap was tunneled through the groin incision underneath the rectus femoris and sartorius muscles ( Fig. 5 ).

**Fig. 6:**
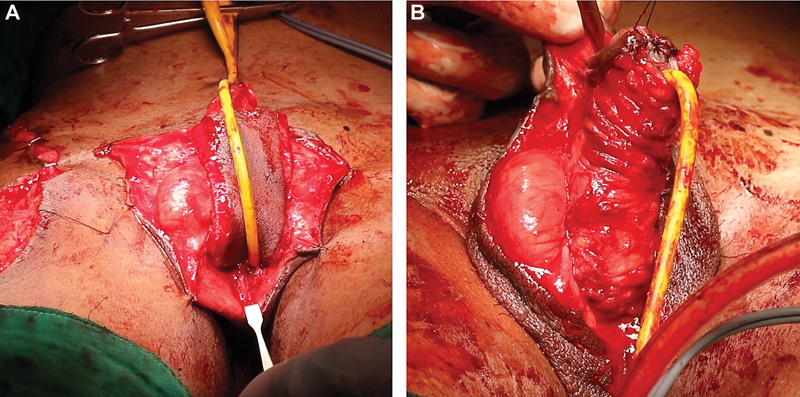
( **A** ) Flap tunneled to the scrotum and lateral edges of the flap deepithelialized. ( **B** ) Flap tubed over a 14-F Foley catheter in two layers. The tubed flap was then fixed proximally to the native urethra with interrupted prolene sutures and distally to the tip of the glans.

**Fig. 7:**
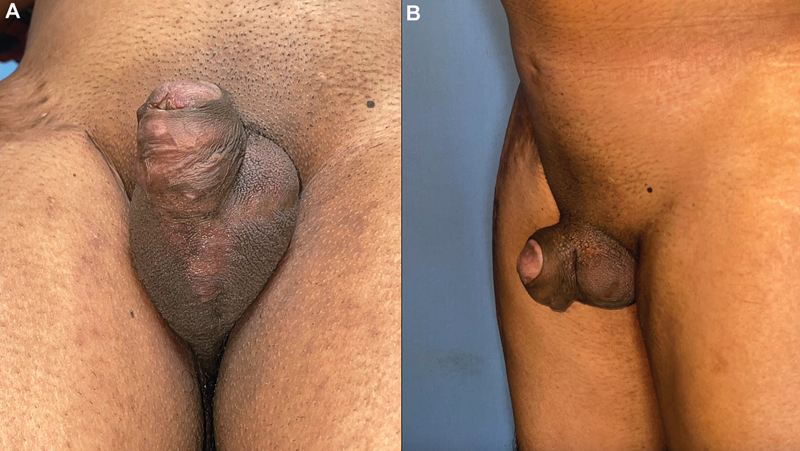
Frontal and lateral views at 1-year follow-up.

The penile shaft and scrotal skin were incised, and the penis was degloved and the scrotal level urethral opening was isolated. The scrotal skin was mobilized, and the prefabricated flap was tunneled underneath it. The peripheral margins of the flap were de-epithelialized. The flap was tubed to create a neourethra by continuous 3–0 PDS sutures ( Fig. 6 ). After completion of the tubing the neourethra was then sutured to the native urethra by interrupted 3–0 PDS sutures; the distal portion was brought in the position and sutured to the tip of the glans. The neourethra was fixed to the native corpora cavernosa to create a single construct and finally the degloved penile and scrotal skin was draped over it and sutured in two layers. The scarred skin was excised but still there was adequate vascular skin available for primary closure. This completed our reconstruction ( Fig. 7 ).

The patient achieved a good functional outcome. No fistula formation was noted postoperatively. The patient was able to micturate in the standing position. The patient is able to have sexual intercourse with his wife and has fathered a child. The penile length in flaccid state is 3.5 cm and in erect state is 7.5 cm ( Video 1A, B ).

## Discussion

The term “hypospadias cripples” describes patients who have had multiple failed hypospadias repair attempts and have significant issues like fistulae, strictures, and scarred penis with limited options for reconstruction. 1 For long segment urethral reconstructions in a hypospadias cripple, we do not have many options available for reconstruction because in these patients the preputial skin has already been utilized for reconstruction and is deficient. 2 The scrotum may be scarred and unsuitable for the introduction of new tissue into the penile shaft. In our patient, there were three urethrocutaneous fistulas with paucity of local tissue. The availability of new lining on the penile shaft is essential to provide enough tissue to form a urethra which can either be a prelaminated microvascular free tissue transfer 3 4 or pedicled flap transfer. 5 6 Khouri et al performed a two-stage technique of total penile reconstruction in which the first stage involved reconstructing the neourethra as a tubed skin graft incorporated in the territory of the lateral arm flap. Second stage performed after 3 to 6 months involved fashioning of the lateral arm flap with its well-epithelialized conduit into a penis.

Our technique of prefabricating the hairless groin skin for making a long 13-cm segment of urethra using the descending branch of LCFA has never been described in literature. Descending branch of LCFA is a long vessel with reliable anatomy. It easily reaches up to the level of groin. 7

The minimum time required for prefabrication is 3 to 4 weeks during which neoangiogenesis and vessel arborization take place, 8 but in our case we waited longer because of patient-related factors which might have made the flap even more reliable. 9 This technique is unique as we don't need preoperative laser treatment because thin hairless groin skin is used, and it eliminates the need of performing a microvascular free tissue transfer with the added repeated flap monitoring required postoperatively. It is extremely reliable and reproducible. It also incurs less financial burden to the patient as it reduces postoperative hospital stay. Our patient was discharged from the hospital on postoperative day 1 after the first stage and on postoperative day 2 after the second procedure.

## Conclusion

Long segment urethral reconstruction using prefabricated hairless groin skin flap based on the descending branch of LCFA is a new technique of near-total urethral reconstruction and to the best of our knowledge has never been described in the literature. It can be used to treat difficult cases of urethral reconstruction for varied indications when there is paucity of local tissues and no other option is available locally, giving us a simple and innovative technique in reconstructive armamentarium.
